# Team climate and job satisfaction in a Mobile Emergency Care Service^*^

**DOI:** 10.1590/1980-220X-REEUSP-2022-0174en

**Published:** 2022-10-03

**Authors:** Hercules de Oliveira Carmo, Marina Peduzzi, Daisy Maria Rizatto Tronchin

**Affiliations:** 1 Universidade de São Paulo, Escola de Enfermagem, Programa de Pós-Graduação em Gerenciamento em Enfermagem. São Paulo, SP, Brazil.; 2 Universidade de São Paulo, Escola de Enfermagem, Departamento de Orientação Profissional. São Paulo, SP, Brazil.

**Keywords:** Prehospital Care, Patient Care Team, Job Satisfaction, Emergency Medical Services, Ambulances., Atención Prehospitalaria, Grupo de Atención al Paciente, Satisfacción en el Trabajo, Servicios Médicos de Urgencia, Ambulancias, Assistência Pré-Hospitalar, Equipe de Assistência ao Paciente, Satisfação no Emprego, Serviços Médicos de Emergência, Ambulâncias

## Abstract

**Objective:**

to identify the team climate and job satisfaction in a Mobile Emergency Care Service (SAMU).

**Method:**

this is a quantitative, exploratory-descriptive study. The sample corresponded to 95 workers, allocated in 40 teams of a SAMU in the Metropolitan Region of the city of São Paulo. We applied three instruments: sociodemographic/labor characterization; Team Climate Inventory; and Job Satisfaction Scale-S20/23, validated for Brazil. Descriptive statistics were used for data analysis.

**Results:**

the total score of team climate was high both among workers (194.5 points; SD = 21) and among teams (197.7 points; SD = 18.4). Satisfaction had a mean total score of 4.5 (SD = 0.4), for workers, and 4.6 points (SD = 0.6), for teams. As for the climate, “Team participation” and “Support for new ideas” obtained agreement percentages >70% in all propositions. Regarding job satisfaction, there was a percentage of concordance >80% in the three dimensions.

**Conclusion:**

the findings show a positive perception of workers about team climate and job satisfaction, supporting SAMU management in promoting a favorable environment for professional practice.

## RESUMO

**Objetivo:**

identificar o clima em equipe e a satisfação no trabalho em um Serviço de Atendimento Móvel de Urgência (SAMU).

**Método:**

estudo quantitativo, exploratório-descritivo. A amostra correspondeu a 95 trabalhadores, alocados em 40 equipes de um SAMU na Região Metropolitana do município de São Paulo. Foram aplicados três instrumentos: caracterização sociodemográfica/laboral; Escala Clima na Equipe; e Escala de Satisfação no Trabalho-S20/23, validadas para o Brasil. Empregou-se a estatítica descritiva para a análise dos dados.

**Resultados:**

o escore total do clima em equipe se mostrou elevado tanto entre os trabalhadores (194,5 pontos; DP = 21) quanto entre as equipes (197,7 pontos; DP = 18,4). A satisfação obteve média no escore total de 4,5 (DP = 0,4), tratando-se dos trabalhadores, e 4,6 pontos (DP = 0,6), referindo-se àas equipes. Quanto ao clima, os fatores “Participação na equipe” e “Apoio para ideias novas” obtiveram percentuais de concordância > a 70% em todas as proposições. Referente à satisfação no trabalho, evidenciou-se percentual de concordantes > a 80% nas três dimensões.

**Conclusão:**

os achados mostram percepção positiva dos trabalhadores sobre o clima em equipe e a satisfação no trabalho, subsidiando a gestão do SAMU na promoção de ambiente favorável à prática profissional.

## RESUMEN

**Objetivo:**

identificar el clima de equipo y la satisfacción laboral en un Servicio Móvil de Atención de Urgencias (SAMU).

**Método:**

estudio cuantitativo, exploratorio-descriptivo. La muestra correspondió a 95 trabajadores, distribuidos en 40 equipos de un SAMU de la Región Metropolitana de la ciudad de São Paulo. Se aplicaron tres instrumentos: caracterización sociodemográfica/laboral; Escala Clima del Equipo; y Escala de Satisfacción Laboral-S20/23, validada para Brasil. Se utilizó estadítica descriptiva para el análisis de datos.

**Resultados:**

el clima de equipo, puntuación total, fue alto tanto entre los trabajadores (194,5 puntos; DE = 21) como entre los equipos (197,7 puntos; DE = 18,4). La satisfacción tuvo una puntuación total media de 4,5 (DE = 0,4), en el caso de los trabajadores, y de 4,6 puntos (DE = 0,6), en el caso de los equipos. En cuanto al clima, los factores “Participación en el equipo” y “Apoyo a nuevas ideas” obtuvieron porcentajes de acuerdo >70% en todas las proposiciones. En cuanto a la satisfacción laboral, hubo un porcentaje de concordancia >80% en las tres dimensiones.

**Conclusión:**

los hallazgos muestran una percepción positiva de los trabajadores sobre el clima de equipo y la satisfacción laboral, apoyando la gestión del SAMU en la promoción de un ambiente favorable para la práctica profesional.

## INTRODUCTION

The work context in mobile pre-hospital care (MPHC) is characterized by specificities that impose challenges on teams, considering the need to prepare to face situations of risks and uncertainties arising from care scenarios and challenges related to the work dynamics and the care network itself^(1)^. Thus, team cohesion, skill and professional competence development, as well as the use of rationality, in order to reduce risks and damages in this area of care, become imperative.

In the Unified Health System (SUS – *Sistema* Único *de Saúde*), the Mobile Emergency Care Service (SAMU – *Serviço de Atendimento Móvel de Urgência*), implemented in 2003, is the central axis of a government strategy for the reorganization and adequacy of emergency care^(2,3)^. Over 18 years, this public equipment has been expanding in the national territory, with improvements to health conditions, providing the population with in situ and immediate care for the most different health problems, causing a decrease in mortality in emergencies^(4)^.

In the current scenario, the organization of work processes in SAMU is configured, mainly, based on care and management dimensions. With regard to care, the activities are concentrated from a structural point of view in: Emergency Regulation Center (ERC), with a work team composed of professionals (physicians, assistant technicians for medical regulation and radio operators), trained in the regulation of telephone calls that require emergency guidance and/or care by the number “192”; classification and prioritization of emergency care needs; and early care for victims after a health problem has occurred, by sending mobile units manned by a trained team (physicians, nurses, nursing assistants and technicians and rescuers), previously communicated by ERC^(2,3)^.

Regarding the management dimension developed by the professionals who occupy the management positions (service coordinator, technical manager and nursing coordinator), the activities are intended for planning, coordination of teams, logistical support, integration of SAMU with other services of the Emergency Care Network (ECN), monitoring of recommended quality indicators, development of educational actions in conjunction with the Emergency Education Nucleus (EEN) and service assessment from workers’ and the population’s perspective^(1,3)^.

Given the above, it is emphasized that teamwork and job satisfaction are phenomena that constitute the practice environment and enhancers for achieving quality and safety care, making it crucial to investigate and understand the singularities that permeate such constructs from the perspective of professionals working in these health services.

Teamwork involves the participation of different professional categories that work together in a close, integrated and interdependent manner. Moreover, they set shared goals, aim to achieve common goals and collaborate with each other to provide health outcomes^(5,6)^. In this regard, the authors point out that teamwork can be analyzed based on team climate^(5)^, defined as “the set of perceptions shared among the members of a team according to policies, practices and procedures that individuals experience in the work context”^(7)^.

Furthermore, job satisfaction is a result of workers’ perception of their work, meeting their work values in line with their needs^(8)^. It consists of an emotional, personal, subjective, dynamic state, and can be modified due to intrinsic and extrinsic conditions of both work and professionals^(9)^.

In the national and international literature, studies have analyzed and pointed out a close relationship between “team climate” and “job satisfaction” in different areas of health care^(10,11)^. However, under the MPHC/SAMU, they are still scarce and represent a considerable gap in scientific knowledge, especially in relation to the management of services and people. Thus, this research aimed to identify team climate and job satisfaction in a SAMU.

## METHOD

### Type of Study

This is a quantitative, exploratory-descriptive study.

### Local

The study was conducted in a SAMU of a municipality in the Metropolitan Region of São Paulo (SP).

### Population/Sample

The study population comprised 185 SAMU workers (physicians, nurses, nursing technicians/assistants, medical regulation assistant technicians, radio operators and rescuer drivers). Of this total, 156 professionals were selected, who worked in the mobile emergency units or in the ERC for a time ≥ to 6 months and who worked in the same team for a time ≥ to 3 months. Those with administrative functions/coordinators/managers and professionals on vacation or on leave from their work activities of any nature during the data collection period were excluded. However, 34 refused to participate, resulting in 122 professionals (total population). Of this contingent, 95 workers (77.8%), members of 40 teams, returned the instruments, composing the sample of this study. Sampling was non-probabilistic and convenience.

Concerning the teams, two worked in the regulation center, made up of auxiliary medical regulation technicians, radio operators and regulatory physicians; 29 in the basic life support unit (BSU), composed of first-aid drivers, nursing assistants and technicians; and nine in the advanced life support unit (ASU), made up of first-aid drivers, nurses and interventional physicians.

### Data Collection

Data collection was carried out between July 12 and September 24, 2021. Three questionnaires were applied, namely: I – professional sociodemographic and labor characterization, II – Team Climate Inventory (TCI), valid for Brazil; and III – Job Satisfaction Scale-S20/23 (JSS-S20/23), valid for Brazil^(12,13)^. The characterization variables were age, gender, training, employment relationship, type of regime and working time at SAMU, working hours per shift, team in which they belong, time working in this team, another employment relationship and immunization against COVID-19, since this last variable interferes with workers’ health and, therefore, with job satisfaction.

Team climate was measured by the TCI, with 38 items distributed in four factors: team participation (12 items); support for new ideas (eight items); team goals (11 items); and task orientation (seven items). The factors consist of affirmative sentences or questions answered using a Likert-type scale. Degrees vary from one to five or from one to seven, and the general score for each factor is obtained by the mean of responses, varying as a function of the number of assertions and degrees. In team participation, the minimum value corresponds to 12 points, and the maximum value, to 60; in support for new ideas, minimum of 8 and maximum of 40; in team goals, minimum of 11 and maximum of 77; and in task orientation, minimum of 7 and maximum of 49. In the total score, the minimum is 38 and the maximum is 226^(12)^.

Job satisfaction was assessed from JSS-S20/23, composed of 20 items, distributed in three dimensions, namely: satisfaction with hierarchical relationships (SHR) (11 items); satisfaction with physical work environment (SPWE) (five items); and intrinsic job satisfaction (IJS) (four items). The assertions are answered based on the five-degree Likert-type scale. From the mean of the sum of the answers, the general score of each dimension was obtained^(13)^.

It should be noted that, in both scales, there is no cut-off point, considering positive/favorable results as the mean values that are closest to the maximum estimated for the overall score and for each factor or dimension.

### Data Analysis and Treatment

The data were entered and organized in an electronic spreadsheet and then processed using the Statistical Package for the Social Sciences (SPSS), version 20.0.

Descriptive statistics were used for the analysis. Categorical variables are presented as absolute and relative frequencies. For numerical variables, summary measures were used (mean, median, minimum, maximum and standard deviation). Team climate and job satisfaction were assessed by the mean scores of professionals and teams, and also rescheduled so that they varied on a scale from zero to 100. Rescheduling consists of reformatting the original distribution of a given scale, in order to shift the values obtained to a common distribution scale so that they have the same order of magnitude, favoring the understanding of the findings.

Scale internal consistency was assessed using Cronbach’s alpha coefficient, in order to verify instrument reliability in the research scenario. In this research, we identified coefficient values greater than 0.80.

### Ethical Aspects

This research was approved by the Research Ethics Committee of the *Escola de Enfermagem* of the *Universidade de São Paulo*, under Opinion 3.890.586, in 2020, and was developed according to the recommendations of Resolution 466/2012 of the Brazilian National Health Council^(14)^. Workers were invited to participate, being informed about the objectives, the investigation method and the guarantee of all ethical issues. All participants signed the Informed Consent Form (ICF).

## RESULTS

Regarding professional sociodemographic characteristics, it was found that the mean age was 44 years (SD = 9.2), ranging from 23 to 61 years. Most declared themselves male 60 (63.2%), and 37 (38.9%) of workers completed higher education. As for the area of training, 40 (64.5%) nursing predominated, with 22 (35.5%) nurses, 17 (27.4%) nursing technicians and 1 (1.6%) nursing assistant.

As for work characteristics, it was found that, regarding the position/function, 38 (40%) acted as rescue drivers, followed by nursing technicians and assistants and nurses, 16 (16.8%), 13 (13.7%) and 9 (9.5%), respectively. It is noteworthy that, although 22 professionals had bachelor’s degrees in nursing, 13 of them performed their activities as nursing technicians or assistants.

Regarding employment relationship, 58 (61.1%) were municipal civil servants, 54 were hired via CLT (56.8%); 53 (55.8%) worked during the day; 85 (89.5%) were working 12X36h/shift; 41 (44.6%) had another job; and 95 (100%) were immunized against COVID-19, 94 (98.9%) with two doses.

The mean working time was 7.9 years (SD = 5.6), ranging from 6 months to 20 years. The time working together in the same team was equivalent to 3.9 years (SD = 4), with variance between 4 months and 14 years.

Regarding team characteristics, a predominance of BSU-type mobile assistance was identified (29; 72.5%), mostly composed of two professionals (30; 75%).

Table 1 presents the results referring to summary measures about team climate and TCI reliability.

**Table 1:** Distribution of summary measures of the Team Climate Inventory factors, total score in the perception of professionals and teams and Cronbach’s alpha value – São Paulo, SP, Brazil, 2021.

Factors	Professionals (n = 95)					Teams (n = 40)					Cronbach’s α
	Mean	SD*	Min**	Max**	Md	Mean	SD*	Min**	Max**	Md	
Team participation	51.6	5.7	38.0	60.0	53.5	52.3	5.3	38.0	60.0	53.8	0.93
Support for new ideas	34.1	4.1	25.6	40.0	34.5	34.7	3.9	25.6	40.0	35.0	0.94
Team goals	66.3	7.8	46.4	77.0	67.5	67.5	6.8	46.4	77.0	67.6	0.95
Task orientation	42.4	5.2	27.0	49.0	43.5	43.2	4.5	27.0	49.0	43.8	0.92
Total score	194.5	21.0	137.2	225.5	198.5	197.7	18.4	137.2	225.5	200.3	0.97

* SD = standard deviation;

** Min-max. = minimum and maximum values; Md = median.

By analyzing the findings of Table 1, it was found that the total TCI score from professionals’ perspective was 194.5 (SD = 21.0) and 197.7 (SD = 18.4) points by teams. With regard to Cronbach’s alpha coefficient, there were general values and between factors higher than 0.90. It is pointed out that the rescheduled results, in the third quartile, presented mean values above 80 points both in relation to the factors and in the general score, indicating high scores between professionals and teams.

In view of TCI’s assertions, “Team participation” and “Support for new ideas” obtained agreement percentages above 70%, highlighting the assertions related to sharing information for and among the team and searching for new ways to analyze problems, and support in the face of new ideas and applicability.

Regarding “Team goals” and “Support for tasks”, the items “How much do you think these objectives are valid for society in general?” (81; 85.3%), which expresses clarity in understanding this aspect, and “Does the team have clear criteria that members try to fulfill to achieve excellence as a team?” (81; 85.2%), stand out with high percentages of assertiveness.

Table 2 shows the summary measures on the perception of professionals and teams regarding job satisfaction and JSS-S20/23 reliability.

**Table 2:** Distribution of summary measures of Job Satisfaction Scale-S20/23 dimensions, total score and Cronbach’s alpha value in the perception of professionals and teams – São Paulo, SP, Brazil, 2021.

Dimensions	Professionals (n = 95)					Teams (n = 40)					Cronbach’s α
	Mean	SD*	Min**	Max**	Md	Mean	SD*	Min**	Max**	Md	
SHR***	4.6	0.5	2.7	5.0	4.7	4.6	0.4	3.7	5.0	4.8	0.92
SPWE***	4.4	0.8	1.6	5.0	4.8	4.5	0.6	2.4	5.0	4.7	0.91
IJS***	4.6	0.6	2.5	5.0	4.8	4.6	0.4	3.5	5.0	4.7	0.90
Total score	4.5	0.6	2.8	5.0	4.8	4.6	0.4	3.3	5.0	4.7	0.95

* SD = standard deviation;

** Min-Max. = minimum and maximum values; Md = median;

*** SHR = satisfaction with hierarchical relationships; SPWE = satisfaction with the physical work environment; IJS = intrinsic job satisfaction.

By analyzing the means of the total score and dimensions, from professionals’ perspective, it appears that the mean total score corresponded to 4.5 (SD = 0.6), with the lowest mean being attributed to the “SPWE” dimension (4, 4; SD = 0.8). As for teams, the total had a mean of 4.6 (SD = 0.4), and the “SPWE” dimension also had a lower mean (4.5; SD = 0.6). Regarding the Cronbach’s alpha value, values higher than those estimated by the authors in total and in the dimensions were identified. The rescheduled results showed that, both in the total score and in the three dimensions, the means were higher than 80 points, obtaining the same score in the third quartile, which indicates a high degree of satisfaction among professionals and teams.

As for the assertions of JSS-S20/23, most were reported by professionals with degrees of agreement above 90% (partial and total), with higher frequencies in the total degree.

In the “SHR” dimension, assertions addressing personal relationships with an instance of power, supervision and support received by superiors obtained percentages above 92 (97%) of agreement. The ways in which negotiations on hiring and benefits are processed showed the lowest degree of agreement (81; 85.3%).

Equivalent to the “SPWE” dimension, high percentages of satisfaction with lighting 90 (94.8%) were verified. However, there was some dissatisfaction with ventilation (11; 11.6%) and air conditioning in the workplace (12; 12.7%).

Regarding the IJS, a higher degree of satisfaction was mentioned in the aspects, portraying work as a provider of achievement, understanding that these workers feel fulfilled, appreciate and enjoy in providing MPHC.

## DISCUSSION

Regarding the sociodemographic variables, the presence of adult professionals was verified, and most declared themselves male. Similar results were evidenced in studies, including SAMU, in Brazil, showing that most professionals were aged between 30 and 45 years^(15,16)^. Internationally, in MPHC services, in Spain, they pointed out that 44.4% were in the age group between 51 and 60 years^(17)^, and in Portugal, they found a mean of 37.2 years (SD = 6, 5), minimum of 25 and maximum of 57 years^(18)^, the latter with a mean lower than that of the present investigation.

Regarding gender, the findings of this research are similar to those found in studies carried out in MPHC services, with a prevalence of men^(16,17,18,19,20)^. Although there is male hegemony in the contingent of professionals, this is not a mandatory condition for working in SAMU. In the imagination of society, symbolically, the type of work in MPHC requires only physical effort to help victims^(20)^.

Regarding education, most had higher education, and the prevalent course among the graduates was nursing, and 13 (59%) of them had a degree; however, they performed activities as nursing technicians or assistants. A similar result was found in a study in a SAMU, in the state of Goiás, where of the 155 nursing technicians, 95 (61.29%) had undergraduate degrees in several areas, especially nursing^(21)^. These findings refer to tensions/contradictions between overqualification of workers and precariousness of work, also present in the context of MPHC services.

In this research, concerning work characteristics, it was identified that the majority worked in the role of rescuer driver (40%), 61.1% were workers with municipal public employment and a CLT work regime (56.8%). The experience time at SAMU obtained a mean of 7.9 years (SD = 5.6) and approximately 100% of workers received two doses of the vaccine against COVID-19, as recommended by the Brazilian National Immunization Program (*Programa Nacional de Imunização*) at the time.

The results regarding position/function were correlated to those found in a national study, involving workers in SAMU, with approximately 30% of rescue drivers^(16)^.

Regarding the employment relationship and regime, it is observed that these depend on the administrative structure, management scope and type, resource distribution and service scope and coverage. Thus, opposite findings were evidenced in SAMU, in the state of Goiás, with a predominance of municipal public servants and statutory regime (60.2%) and working time between 4.7 and 6.5 years^(21)^. In the international scope, a study showed that the time of experience of professionals corresponded to the period between 5 and 15 years^(18,22)^.

Regarding the teams, most worked in BSU, composed mainly of two professionals. The time working on the same team was approximately 4 years. Research that analyzed the SAMU implementation process in 2015, revealed that, regarding the distribution of mobile units provided for in legislation^(2,3)^, Brazil had a contingent of 2,643 BSU, 574 ASU, 224 motorcycles and 7 aeromedical personnel. Despite the expansion of the service, it is necessary to pay attention to the need for an increase in the number of ASU, given the relevance of this type of service and the qualified workforce available^(23)^.

The time working in the team was investigated in investigations, specifically in PHC, identifying a mean of 61.83 months^(10)^ and 42.9 months^(24)^, approximately 3.5 and 5 years, respectively. The longer working time in the team contributes to effective work, as it encourages interaction, sharing and joint decision-making; however, the time should not be too long, as this situation tends to create relationships of familiarity^(10)^.

Regarding team climate, from professionals’ and teams’ perspective, the results showed mean and median values higher than those found in studies conducted in Primary Health Care (PHC) in the state of São Paulo. Thus, regarding the total score, the mean was 167.9 (SD = 29.3) and the median was 167.0 points^(24)^, and when rescheduled, the results indicated a mean total score of 76.2 points^(10)^. Regarding the four TCI factors, the findings identified in the present investigation were also superior to those found in these studies^(10,24)^.

When approaching team climate, it is possible to associate it with a photographic representation, once it expresses how its members feel comfortable participating and giving their opinion in decision-making, how they are receptive to new ideas, reflect on work and outline common goals. Such characteristics encourage the execution of activities with creativity as well as the construction of different ways of caring in partnership with the community, patients and other health care services^(10)^.

Due to high percentages in “Team participation” and “Support for new ideas”, it is possible to infer that professionals from the studied SAMU share information, look for different ways to analyze the problems, support the new ideas proposed among them and try to apply them in the practice environment.

In MPHC, communication permeates the entire dynamics of the work process, passing through regulation, care and management center, and the relationship with patients/users. Therefore, it is vital that the information shared is objective and clear so that teams recognize the situations and prepare to assist effectively^(25)^.

When analyzing teamwork, authors highlight that communication plays a central role and, when effective and of quality, enables the articulation of actions, interaction and cooperation among professionals^(6,10)^. Moreover, interaction becomes an important agent of the work process, as it allows the exchange of information between the team and makes it possible to direct the efforts of professionals to solve problems, in addition to encouraging collaboration to achieve goals and contribute to a safe environment and the development of ideas and actions capable of generating innovation at work^(24,26)^.

Regarding “Team goals”, it can be considered that, from participants’ perspective, goals are valid for them, for the team and society; however, greater clarity and understanding are needed so that they are actually achievable. Thus, outlining common objectives is considered one of the main components of a team’s effectiveness, since it refers to its interest, involvement and commitment, impacting results such as professionals’ and patients’ satisfaction^(12,26)^.

An investigation in a SAMU, in southern Brazil, revealed that ERC professionals expressed feelings of guilt in situations in which an ambulance was not sent in a timely manner for care, especially when the outcome resulted in death. This fact is also detrimental, since it generates tension and prevents achieving institutional and work objectives^(27)^.

Positive perceptions regarding “Task orientation” were pointed out by SAMU professionals, showing that the teams have clear criteria for achieving results. Notably, a favorable team climate requires responsibility and commitment from individuals and teams to perform tasks/activities with excellence^(7,24)^.

Considering the scores (total and dimensions) obtained in terms of job satisfaction, it was found that both professionals and teams are strongly satisfied in the SAMU of this investigation. These findings corroborate the international investigation, which pointed out high job satisfaction rates of professionals working in MPHC^(20)^services. In the national literature, the result of a study in SAMU was antagonistic^(21)^.

The findings of this research were also superior when compared to investigations developed in other areas of health care, such as PHC (mean of SHR 3.4 points SD = 0.8; SPWE 3.5-SD = 1.0; IJS 3.8-SD = 0.6)^(28)^ and hospital institution (total score with a mean of 3.22 points SD = 0.89; IJS 3.30-SD = 1.03; SPWE 3.13-SD = 1.08)^(29)^.

Research conducted with Family Health Strategy teams with oral health, in the city of São Paulo, using JSS-S20/23 rescheduling, found that the mean total score was 75.05 points, with 75.24 points in the “Intrinsic job satisfaction and physical environment” dimension and 74.78 points in the “SHR” dimension^(10)^.

In view of what has been mentioned, it appears that both in the total score and in the dimensions, the mean values of the aforementioned studies varied between 3.1 and 3.8, and when rescheduled between 74.78 and 75.24, they presented results lower than those of this research, whose means were higher than 4.4 and 88.3 points.

Regarding the JSS-S20/23 assertions, positive perceptions regarding “SHR” demonstrated professionals’ satisfaction with the relationships established with their managers/supervisors. On the other hand, there was dissatisfaction with issues about negotiations and benefits and autonomy. In this sense, a study measuring job satisfaction at a SAMU in the state of Goiás found that relationships with supervision were the attribute with the highest mean and highest percentage of satisfaction^(21)^.

As for the “SPWE” dimension assertions, results similar to those of this research were obtained, with air conditioning and ventilation of the environment, plus lighting, hygiene and health conditions as the main generators of dissatisfaction among nursing workers^(16)^.

Regarding the IJS, the proposition with the highest satisfaction score referred to work as a provider of achievement, understanding that these workers feel fulfilled, appreciate and enjoy providing assistance. These findings corroborate other studies that also obtained higher percentages in this dimension^(18,28)^.

A study should be highlighted, reporting that benefiting a person who needs MPHC is essential for the team, and the misfortunes that affect victims give meaning to work, since the activities carried out aim to alleviate suffering or save life. Furthermore, it is not uncommon for SAMU professionals to express that the greater the complexity and demand for care, the more pleasant the work becomes and that feeling useful, motivates them to face the challenges of each occurrence^(30)^.

It is understood that the limitations of this study lie in the sample size and type of team, mostly composed of two professionals. The discussion may have been restricted, given the lack of research using TCI and JSS-S20/23, in the context of SAMU, in the national territory and team climate investigations are carried out, especially in PHC. As for job satisfaction, most studies occur in other areas of health care using different instruments, making it difficult to measure and compare the results.

## CONCLUSION

In the perception of professionals and teams, there is a team climate with high scores (total and factors), therefore, favorable to teamwork, with emphasis on “Team participation” and “Task orientation”, showing high satisfaction at work, especially in the SHR and IJS dimensions. Therefore, it is necessary to invest in improvements related to the physical environment, especially regarding ventilation and air conditioning.

Given the extensive literature review carried out, this is pioneering research on the themes of teamwork climate and job satisfaction in SAMU using TCI and JSS-S20/23 in the national territory. It is believed that the findings constitute elements intended to support SAMU management, in order to promote and foster a positive climate for teamwork, provide job satisfaction and qualify care within the MPHC.

Finally, it is recommended to produce investigations applying TCI and JSS-S20/23 in different SAMU, as well as research using qualitative approaches and mixed methods, with a view to broadening the understanding of team climate and job satisfaction phenomena in this health care model.
